# Dependency of Queensland and the Great Barrier Reef’s tropical fisheries on reef-associated fish

**DOI:** 10.1038/s41598-020-74652-2

**Published:** 2020-10-20

**Authors:** Christopher J. Brown, William Taylor, Colette C. C. Wabnitz, Rod M. Connolly

**Affiliations:** 1 Australian Rivers Institute – Coast and Estuaries, School of Environment and Science, Griffith University, Nathan, QLD 4111 Australia; 2 Institute for the Oceans and Fisheries, The University of British Columbia, 2202 Main Mall, Vancouver, BC V6T1Z4 Canada; 3 Center for Ocean Solutions, Stanford University, 473 Via Ortega, Stanford, CA 94305 USA; 4 Australian Rivers Institute – Coast and Estuaries, School of Environment and Science, Griffith University, Gold Coast, QLD 4222 Australia

**Keywords:** Climate-change ecology, Marine biology

## Abstract

Coral reefs have been subject to mass coral bleaching, potentially causing rapid and widespread degradation of ecosystem services that depend on live coral cover, such as fisheries catch. Fisheries species in tropical waters associate with a wide range of habitats, so assessing the dependency of fisheries on coral reefs is important for guiding fishery responses to coral reef degradation. This study aimed to determine how fisheries catches associate with coral reefs in Queensland, Australia. Queensland’s largest fisheries did not target fish associated with reefs, but specific sectors, particularly aquarium fisheries and commercial fisheries in the mid to northern region had a high dependence on species that use coral reefs. Regions that had a greater relative area of coral reefs had higher catches of species that depend on live coral, suggesting that coral area could be used to predict the sensitivity of a jurisdiction’s fisheries to bleaching. Dynamic analysis of stock trends found that coral trout and red throat emperor, the two largest species by catch for the reef line fishery, were at risk of overfishing if habitat loss caused declines in stock productivity. Management of fisheries that are highly dependent on reefs may need to adapt to declining productivity, but further research to support ongoing reforms in Queensland’s fisheries is needed to quantitatively link reef degradation to stock production parameters is needed.

## Introduction

Global coral bleaching events are occurring with increasing frequency^1^ and can cause widespread coral death^2^. Recently, mortality associated with mass bleaching events has become a major contributor to the degradation of coral reefs across areas as large as the Great Barrier Reef^3,4^. Living coral reefs are structurally complex habitats that support a high biomass and diversity of fisheries species, including finfish, crustaceans, molluscs and echinoderms. Coral mortality can cause changes in the composition of reef fish communities^5^ and affect the composition of fisheries catch^6^. Coral-associated fisheries can form an important component of many tropical fisheries, supporting food and nutritional security, livelihoods, cultural services and economies^7,8^. The productivity of tropical fisheries is therefore threatened by mass coral mortality events^9–11^. Widespread loss of fishery habitats may require re-assessment of fisheries management policies to account for changes in productivity and enable stocks to recover from environmental pressures^12^.

The Great Barrier Reef (GBR) in Queensland, Australia, was recently subjected to two consecutive summers of mass bleaching events (in 2016 and 2017^13^), raising concern that fisheries may be affected^5^. In addition to bleaching, perhaps one of its most visible impacts on reefs, climate change is having a multitude of other effects on tropical fisheries, including via direct effects of heatwaves on fish populations^4,14^, the effects of sea level rise on coastal vegetated habitats^15^, cyclones^16^ and through changes in coastal circulation^17^ and coastal water quality^18^. However, the climate threat that coral reefs face is particularly widespread, so may affect ecosystem services simultaneously across large scales. It has therefore inspired recent management responses including coral restoration (e.g. through the Great Barrier Reef Fund^19^) and actions aimed at enhancing coral recovery through improvements in water quality^20^. Coral recovery may benefit tropical fisheries, but these fisheries also target a large range of other species that associate with other tropical habitats e.g.^21,22^. In Queensland, commercial fisheries include line fisheries targeting meso-predators directly over coral reefs; diving for lobster and sea cucumber on coral reefs; trawling for prawns and scallops over soft-sediments; and line fishing for pelagic predators^23^. Indigenous, charter and recreational sectors also operate across a variety of habitats from inshore seagrass meadows and estuaries to offshore coral reefs^23,24^. This diversity of habitats and species suggests that tropical fish catches may have decoupled responses to environmental change, potentially enhancing the resilience of the state’s fisheries to reef degradation e.g.^25^. Therefore, the benefit of coral recovery actions for tropical fisheries needs to consider the fraction of fish catch that associates with reefs^9,26^.

Past studies have revealed variable effects of bleaching and coral loss on fished species and their fisheries^6,11,27^. In Queensland, the fisheries sectors most likely to be sensitive to coral loss are the reef line fishery and the aquarium fishery. This sensitivity of this aquarium sector is well recognized, because catches include live coral^28^, as well as a range of fish species that strongly depend on live corals^29,30^ and anemones (which also bleach)^31^. The aquarium fishery has a coral stress response plan that is widely endorsed by the fishing industry^32^. The reef line fishery primarily catches coral trout (*Plectropomus and Variola spp.*), lutjanids and lethrinids, and its management has also considered the impacts of coral loss^33^. Ecological responses of fished species to coral loss are variable. For instance, a time-lag of > 5 years is expected between coral mortality and changes in fishery productivity^4,11,27^ and there is no short-term response of coral trout abundance indices to bleaching events^33^. Some species, particularly herbivores, can also increase in abundance following coral mortality events and fisheries respond by changing the size and composition of catches^6,27^. The first step to anticipating these complex responses in a tropical fishery as a whole is therefore to quantify how much and which components of the fish catch associate with coral reefs and live coral.

In this study, we review coral habitat usage for species targeted by commercial, recreational and charter fisheries in the state of Queensland, Australia. We then assess their dependence on coral reefs by region and sector. We aim to identify the fishing sectors and regions that might be sensitive to change following widespread coral loss. The diversity of tropical fishery species means it is important to consider variation in habitat use of fishery species across sectors and region. Identifying whether a jurisdiction’s fisheries are sensitive directly to the loss of live corals and indirectly via reef degradation is important for setting strategic fishery management advice and to enable forward planning for potential changes in the productivity of fisheries that supports the sustainability of catches.

## Methods

### Fish habitat associations

Fish-habitat associations were first assembled from the global Fishscape database (Brown et al. 2019, available at https://www.seascapemodels.org/fishscape/). Additional information focussing on Queensland species was added through a targeted review of published literature. For each of the six major gear types (pot, otter trawl, net, line, dive, beam trawl) we ranked species and species groups in order of highest to lowest catch from 2008 to 2017 (by Australian Financial years, which end 30th June each year). We then documented habitat associations for the species and species groups that made up 95% of catch (by weight) for each gear type. This process resulted in habitat associations for 57 commercially targeted species and species groups, including finfish, chondrichthyans and invertebrates, collectively referred to here as ‘fish’.

Each data entry includes the species, life-stage, observation method (direct observation, catch survey, e-tags, video survey, diet analysis or experiments) and habitat type(s) the species was observed to associate with. Habitats were first categorised as physico-chemical (e.g. a certain salinity range) or substrate type. Substrate types were grouped into coral reefs, rocky reefs, seagrass, mangroves, macroalgae, and unvegetated soft-substrate. Records used in our analyses were extracted from 131 publications for a total of 1043 database rows.

As a key component of this study, an additional field was added to Fishscape that defined increasingly dependent levels of association with corals, namely:

1. Not known to be associated with coral reefs (live or dead);
2. Associated with coral reefs (which may include a mix of live corals and other habitats), but not known to be dependent on live coral cover;
3. Known dependence on live coral cover;
4. Obligate dependence on live coral cover.

These categories were chosen to reflect the potential sensitivity of a species to coral death and to some extent degradation of the habitat structure that is provided by live corals. Barramundi (*Lates calcarifer*), for example, was classified in Category 1, because the species primarily occurs over estuarine soft-sediment and in vegetated habitats^34^. Category 2 included species known to occur on reefs, but that do not directly depend on corals. Many of these species also associated with other habitats. The curryfish, *Stichopus hermanni*, for instance was placed in Category 2 because, although it associates with reef habitat^35^, we found no evidence that the species is directly dependent on live coral. Impacts of live coral loss on species in Category 2 are likely to be weak, for instance some mackerels (*Scomberomorus* spp.) that use multiple habitat types. A species was classified as Category 3 only if specific evidence pointed to its dependence on live coral cover. If published literature indicated that a species associated with coral reefs, with no specific evidence suggesting that it required live coral habitats, it was placed in Category 2. The coral trout, *Plectropomus maculatus,* for instance, was placed in Category 3 because there is specific evidence that it has higher recruitment to live coral habitat than to non-coral microhabitats within reefs^36^. Obligate dependence on live coral (Category 4) was difficult to determine. The only case of obligate dependence we found was for select species caught by the aquarium industry. However, available fishery data was not always reported at the species level. Where species were grouped in catch data, their category was conservatively assigned the highest category of a species within that group. Thus, for the aquarium sector, this meant that catches listed as being from the Chaetodontidae family (butterflyfish), but without species level identification, were classified in Category 4, because some Chaetodontidae feed on live corals^37^—acknowledging that many species targeted by the industry do not feed on live coral because these would do poorly in captivity^29^.

### Data and analysis for Queensland's fisheries

Data on Queensland’s commercial fisheries were obtained from logbook records via the QFish online portal^23^ (further details provided in supplementary material). The data included weight of catch from the major commercial fisheries. We analysed mean catches over the decade 2008–2017. Much of the data extracted from QFish was recorded at higher taxonomic levels (or for quite arbitrary categories, like ‘squid’), rather than species level. The group ‘fish’, which accounted for < 1% of the state’s total catch, was excluded from analyses because it was too broad to be useful.

In the next step data was extracted from QFish for species groups by Queensland's fisheries reporting regions and species groups by gear types. The reporting regions (known as ‘Mapstone regions’) from north to south were: Eastern Torres Strait, Gulf of Carpentaria, Far Northern, Cairns, Townsville, Mackay, The Swains group, Capricorn Bunker and Sub-tropical. The habitat associations were then matched by region and gear to identify regional and gear-specific differences in habitat use of targeted species.

Next, we posited that a region’s percent catch for coral associated/dependent species would be predicted by the relative area of coral reefs within that region. If true, this relationship may inform on coral habitat use in other jurisdictions, at least where fisheries are predominantly commercial. Accordingly, percent commercial catch that fell into each category was regressed against the percent coral reef area in each Mapstone region. The latter was derived from the area of submerged reefs (data from^38^) and does not account for % live coral cover. Regression analyses (Bayesian linear models with a Gaussian likelihood) were estimated with the Integrated Nested Laplace Approximations with the R INLA software package^39^. The default broad priors were used (improper flat prior for the intercept, Gaussian with mean zero and variance 1000 for the slope, and log-gamma on precision for the standard deviation).

### Data and analysis for hand-capture fisheries

The hand-capture fisheries are divided into sea cucumbers and the aquarium sector. Data for commercial sea cucumbers (called ‘harvest’ fisheries by Queensland fisheries) were available as annual catches by species, as wet weight (reported as dry weight and then back converted by Queensland Fisheries to wet weight), aggregated across the whole of Queensland. Data for aquarium fisheries were available as number of individuals collected per year by broad taxonomic groups at a regional level. Data on coral collection was also available as number of pieces collected in 2016–2018. Certain group classes were assigned very high levels, taxonomically speaking (e.g., ‘crustaceans’). Therefore, aquarium taxa were assigned sensitivity to coral loss categories according to a slightly different methodology. Their sensitivity was classified according to previous studies that have comprehensively assessed the dependence of coral reef fish on live corals globally^37^ and for parts of the Great Barrier Reef^5^. All aquarium species fell into Categories 2–4. Amphiprion species (anemonefish) were also included in the high vulnerability category (4), because they are sensitive to bleaching of their host species^31^. All coral was considered category 4^28^. Results are presented for catches across the five Mapstone regions where the fishery operates, but we excluded coral pieces from these graphs.

### Data and analysis for charter and recreational fisheries

We analysed charter fishery data in a similar manner to the commercial data, because it is reported as weight per species per year per region in logbooks. The recreational data we accessed through Qfish was collected through a phone call survey^40^. Data from surveys conducted in 2010^24^ and 2013^41^ were combined to represent the catch of coral-associated species by recreational fishing regions.

### Risk of overfishing in reef fisheries subject to sudden productivity declines

We quantified the risk of overfishing for coral-dependent fish stocks if subjected to a sudden productivity decline, such as would be expected from coral loss and reef degradation. For this dynamic analysis, we estimated maximum sustainable yield from time-series of CPUE for the six commercial food-fish groups we found to be most dependent on coral reefs: coral trout, red throat emperor (*Lethrinus miniatus*), saddletail snapper (*Lutjanus malabaricus*), red emperor (*Lutjanus sabae*), goldband snapper (*Pristipomoides multidens* and *typus*) and redspot king prawn (*Melicertus longistylus*) (Table S1). Some, but not all, of these species groups have current stock assessments, so for consistency we conducted our own analysis of stock status using CPUE trends.

We estimated the MSY because it is a commonly used reference point for classifying stock status^42^. Current status was determined by fitting Bayesian surplus production models to each CPUE time-series (details in supplementary material^43^). We chose to use surplus production models, because previous analyses have used them successfully to study the impacts of environmental change on Queensland’s fisheries^44^. We modified earlier approaches to allow a change in the catchability parameter in 2004. 2004 was the year of the rezoning of the GBR and a restructure of the line fishery, it is likely both events had significant impacts on fishing efficiency (supplementary material). We also ran a sensitivity analysis to account for possible technological changes where we assumed an increase in fishing efficiency over time of 1% per annum.

We could then quantify the posterior probability that current catch exceeded the MSY estimate. We quantified the risk of reef habitat loss as the probability that current catch would exceed MSY given a sudden reduction in the intrinsic growth parameter (r). A decline in the intrinsic growth is representative of the loss of reef habitat, because reef habitat supports recruitment of reef species^12^. We varied the per cent reduction in the intrinsic growth from 0 to 100% in 1% increments, then calculated the probability that the average of catches from 2016 to 2018 would exceed the MSY.

## Results

### Commercial fisheries

**Figure 1:**
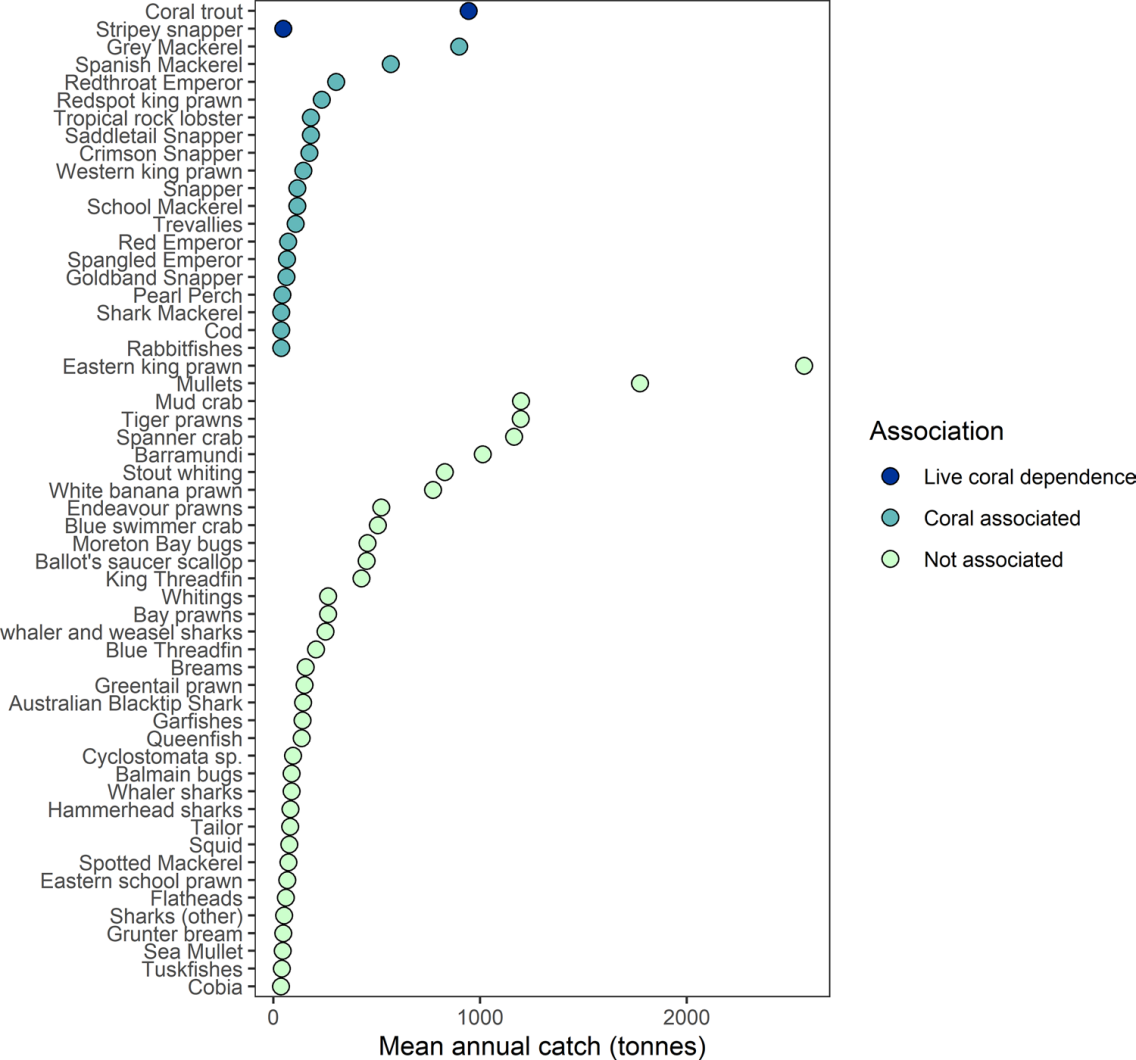
Mean annual catch (tonnes) for Queensland's commercial fisheries (excluding hand capture fisheries) landed between 2008 and 2017, with taxa rank ordered by sensitivity to coral loss categories, then by catch. A complete list of species is provided in Table S1. Live coral dependence (Category 3), Coral associated (Category 2), Not known to be associated with coral reefs (Category 1). There was no commercial catch in Category 4 (obligate dependence).

In linking the fish-habitat data to total commercial fish catch, we found that in Queensland, catches were dominated by prawns and estuarine species that do not associate with reefs. Specifically, we found that between 2007 and 2017, 78% of catch showed no evidence of association with coral reefs (category 1), 17% was from species known to associate with coral reefs (Category 2), 5% was composed of species with documented dependence on live coral cover (Category 3), and there was no reported catch for species with obligate dependence on live corals (Category 4) (Fig. 1). The primary species group which had documented dependence on live coral was coral trout (*Plectropomus spp.* and *Variola spp.*), which is caught in the line fishery.

**Figure 2:**
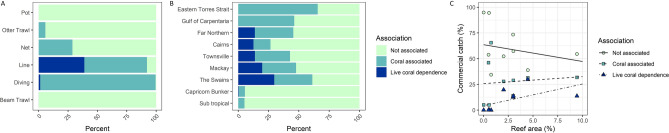
Proportion of commercial catch (2008–2017) falling into the first three categories of coral association / dependence by (**A**) gear and (**B**) sector. Regression of percent of fish catch falling in the first three sensitivity categories against percent area of corals (**C**). Live coral dependence (Category 3), Coral associated (Category 2), Not known to be associated with coral reefs (Category 1). There was no catch in Category 4 (obligate dependence).

The sectors with the highest proportional catches of coral associated species were diving and line fishing, which primarily target tropical spiny rock lobster (*Panulirus ornatus*) and *Plectropomus spp.* as well as lethrinids, respectively (Fig. 2A, Table S1). The line fishery caught some species that were not coral associated, in particular some shark species and spotted mackerel (*Scomberomorus munroi*). Other sectors primarily caught species with no evidence of association with coral reefs. Most of the catch in the dive fishery was obtained from the far north. Most of the line fishing catch was derived from the mid-coastal region, where coral associated or dependent species made up a majority of the catch (Figure S1). The pot fishery that primarily targets inshore crabs, and the beam trawl fishery that primarily targets prawns, had < 1% catch reported to be associated with reefs. Note that the ‘fish trawl’ sector (not shown here because data were incomplete) would also likely have low sensitivity to coral loss, because it primarily targets stout whiting (*Sillago robusta*), a species not known to associate with coral reefs.

Coral associated or dependent species were caught in all regions and accounted for > 40% of catch in 6 out of 9 regions (Fig. 2B). The Torres Strait was characterised by a high proportion of coral associated catch, because fishing in this region is dominated by line fishing and diving. The Far northern region also has line fishing, but had a smaller percentage of reef associated species than Torres Strait, because of a large catch of prawns. The moderately high rates of coral-association for species caught in The Swains group, Townsville and Mackay regions were due to high catches in the line fishery. Commercial fisheries in Capricorn Bunker and sub-tropical regions had the lowest proportions of coral associated catch given that target species were mostly not reef-associated (e.g. prawns and *Sillago spp.*).

Spatial correlations between fisheries catches and the relative area of coral reef habitat showed different relationships for different categories (Fig. 2C). The percent of fish catch for species not associated with corals was weakly negatively related to percent area of coral reefs (Fig. 2C, median slope estimate =  − 1.64 with a 0.77 probability that the slope was < 0). The catch of coral-associated species was not related to the area of coral reefs. The catch of species dependent on live corals increased weakly with the percent area of coral reefs (median slope estimate = 2.1 with a 0.88 probability that the slope was > 0).

### Hand capture fisheries

The sea cucumber fishery had an annual average harvest between 2008 and 2017 of 311 tonnes. All sea cucumber taxa had evidence of potential association with coral reefs. No evidence was found pointing to the specific dependence of sea cucumbers on live coral cover and many of the associations between sea cucumber and coral may be weak. For instance, *Holothuria scabra* is often found on soft sediment habitats near to reefs, but there is limited evidence for a direct dependence on corals other than a preference of larvae to settle on crushed dead coral rather than sand^45^.

**Figure 3:**
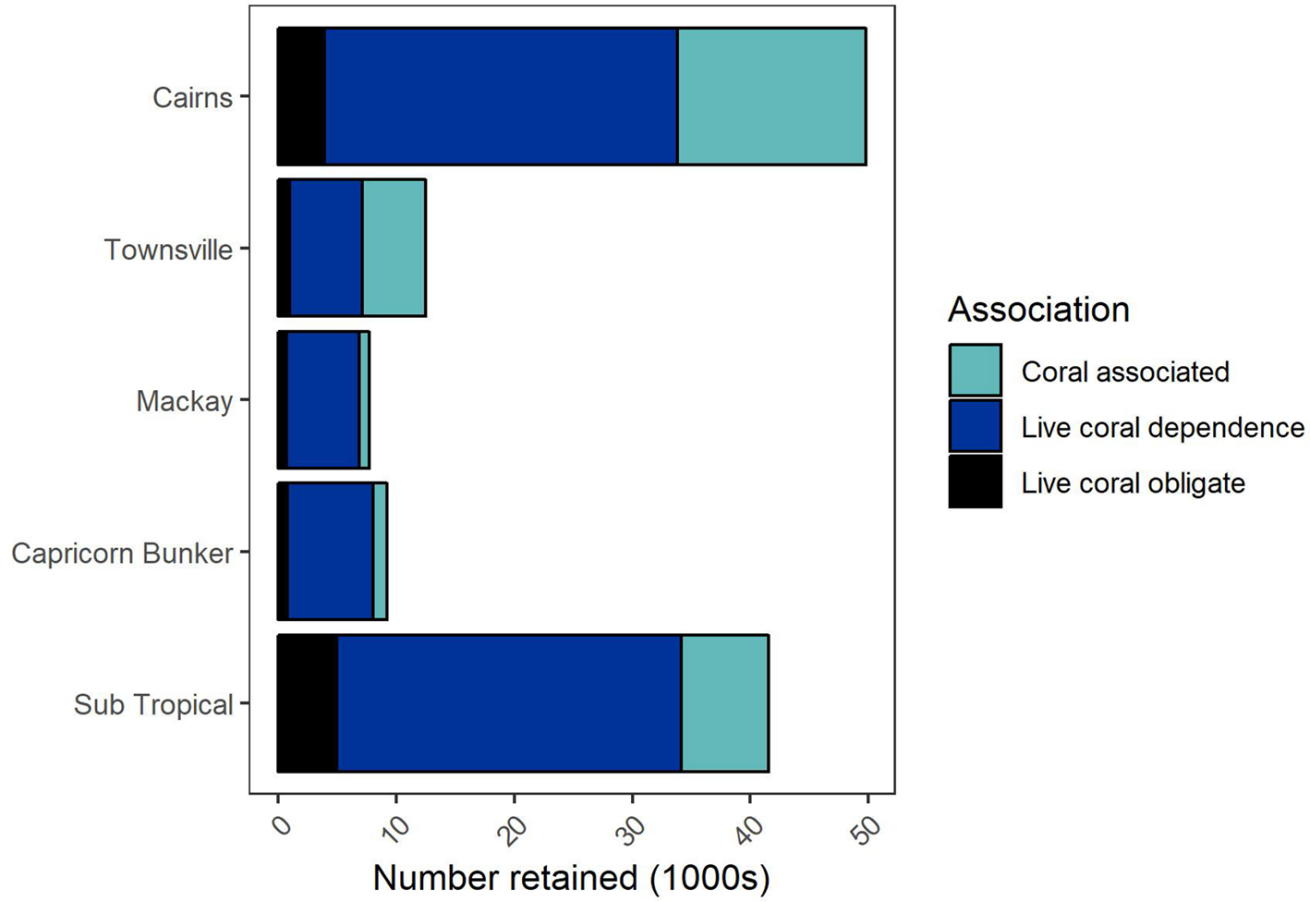
Number of individuals retained by the aquarium fishery by reporting regions and coral association categories. Quantities are means over annual harvest for the period 2008–2017. Coral associated, live coral dependence and live coral obligate dependence correspond to categories 2, 3 and 4 respectively in the text.

Numbers of harvested aquarium specimens varied by region and sensitivity category (Fig. 3). The highest catches were reported from the Cairns region, which has experienced bleaching, and the sub-tropical region where bleaching has only been patchy (Fig. 3). Fishers in both these regions also collected the greatest number of taxa in Categories 3 and 4. Relative to total catch, the northern regions of Cairns and Townsville had the greatest proportion of taxa in the categories for dependence or obligate dependence on live corals (40% and 50% respectively), though we note that the high percent of coral obligate species may reflect the poor taxonomic resolution of data and our conservative approach to classification, rather than large catches of corallivores. Coral collection occurred primarily in northern regions with the majority of catch coming from Townsville (32%) Mackay (34%) and Cairns (19%).

### Recreational and charter fisheries

**Figure 4:**
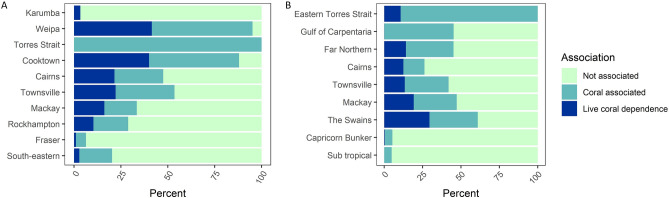
Recreational fishery (**A**) and charter fishery (**B**) catches by region and coral association categories. Values for the recreational fishery are based on number of individual fish, and by biomass for charter fisheries. Reporting regions differ slightly between the two fisheries.

For recreational fisheries, 8.7% and 3.8% of total catch numbers was made up of species associated with coral reefs or species known to be dependent on live coral, respectively. For charter fisheries, 17% of total catch by weight consisted of species associated with coral reefs and 7.4% of species with dependence on live coral. Recreational and charter fisheries had a similar pattern of regional sensitivity to coral loss as commercial fisheries, with greater catch of coral-associated fish harvested from mid-coast and north coast regions, like Cooktown, (Eastern) Torres Strait, The Swains, Mackay, Townsville and Cairns. These regions have coral reefs that are readily accessible by boat, and catch was dominated by a mixed assemblage of coral reef fish, including coral trouts, lutjanids and lethrinids. Weipa (Gulf of Carpentaria) had the highest relative recreational catch of coral associated fish (54% and 42% for coral associated and coral dependent, respectively, Fig. 4). The high catch of coral associated species in Weipa, a region dominated by estuarine and soft-sediment habitats, may be due to the low taxonomic resolution of catch data from Weipa. For instance, 39% of the Weipa catch was classified as ‘Cod & Groper—unspecified’.

### Dynamic analysis

**Figure 5:**
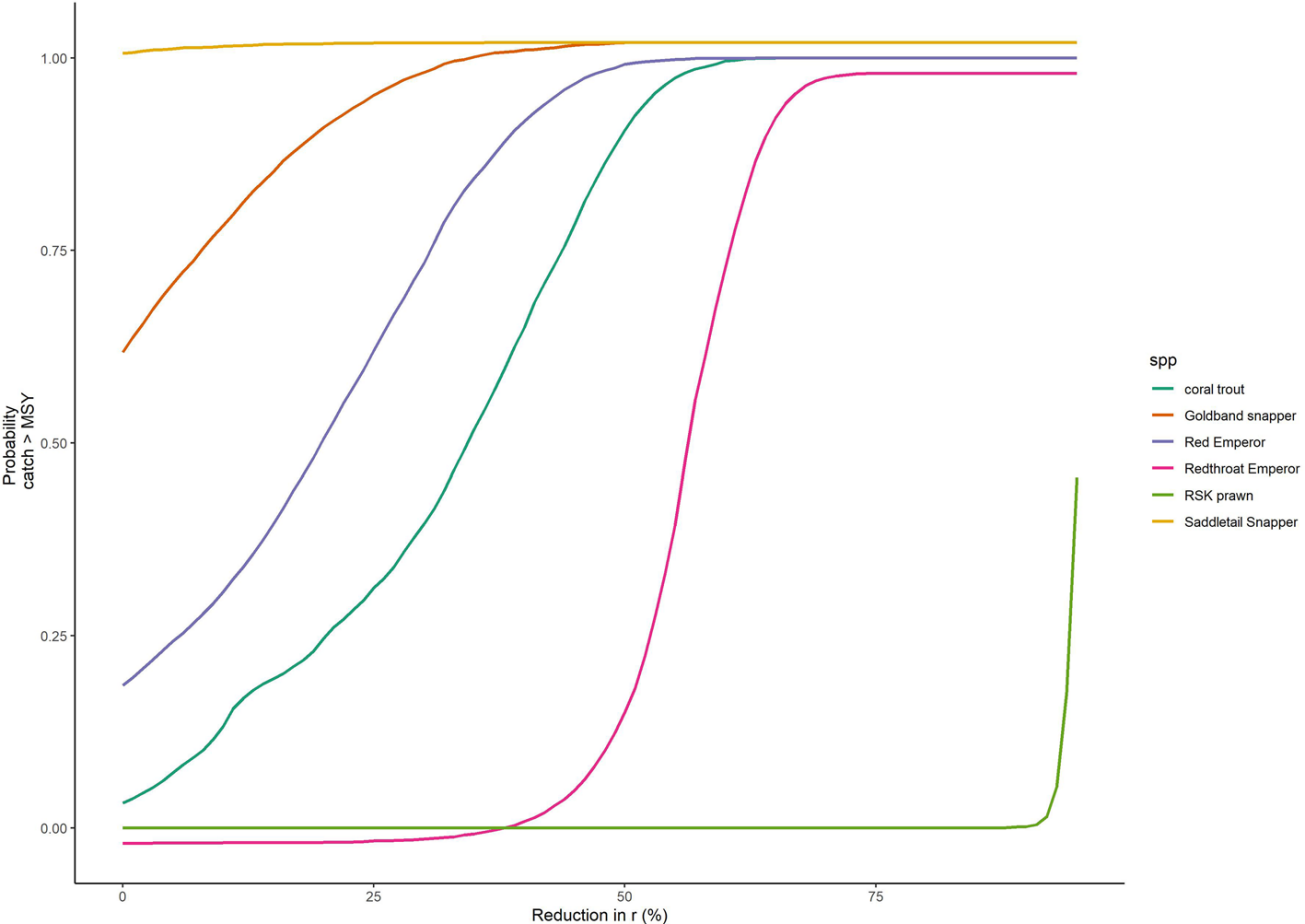
Probability present day catch exceeds estimated MSY reference point given different assumed % reductions in the intrinsic growth rate (r).

The surplus production model fits were reasonable for most species (R^2^ > 0.8, Table S3). For coral trout, the predicted biomass as a per cent of unfished biomass in 2017–2018 was consistent with a recent stock assessment: 95% credible intervals were 47–87% (median 67%) of unfished compared to 68% in^46^. For most species the credible intervals on the catchability coefficients before and after the 2004 restructure had broad overlap, but catchability for red throat emperor was predicted to have declined considerably (Table S4). The surplus production model found that catches for all species except saddletail snapper and goldband snapper were likely below the MSY presently (Fig. 5, left side with 0% reduction in r).

Goldband snapper and red emperor had a heightened risk of overfishing if productivity had a sudden decline of > 20% (> 50% risk for overfishing). Coral trout was predicted to be at risk of overfishing for declines in productivity of > 30%. The risk of overfishing was lowest for red throat emperor and red spot king prawn. The low risk of red throat emperor being overfished, despite its low productivity parameter, can be explained by the predicted decline in catchability after 2004. Stocks were slightly more at risk of overfishing if we assumed a 1% efficiency increase per annum, but the order of stocks in their risk of overfishing was the same as assuming no efficiency increase (Figure S2).

## Discussion

### Coral dependence of Queensland's fisheries

Queensland’s catch consists predominantly of species not associated with reefs. This is not surprising as Queensland’s largest fisheries target prawns as well as coastal and estuarine fish and crustacean species. Thus, most of the species these fisheries target do not associate with reefs.

The aggregate statistic on reef-associated catch belies regional and sectoral variation in sensitivity to coral loss. Four sectors were found to be most dependent on coral reefs: the line fishery, which primarily operates around coral reefs, the dive fishery for lobster, and fisheries targeting sea cucumber and live aquarium species. This pattern across sectors manifested as variability in regional dependency on coral reef habitats. For instance, a high proportion of the catch in the Eastern Torres Strait is from the tropical rock lobster fishery, so this region is likely to be more sensitive to coral reef degradation. Similarly, the Swains region (reefs offshore of Rockhampton and Mackay) had a high proportion of coral-associated and coral-dependent species in catches because of the predominance of line fishing. Other northern regions, like Cairns, had lower proportions of catches of coral-associated species, not because they caught less coral-associated fish, but because a large proportion of the catch comes from trawl fisheries. Live captures in the aquarium fishery consisted of a high proportion of coral-associated and dependent fish species, including coral itself, and this fishery derived a large proportion of its catch from reefs in northern regions that have recently experienced severe bleaching^13^. Thus, this fishery is likely more sensitive to the effects of coral bleaching than most of Queensland’s other commercial fisheries. Recreational and charter fisheries also took a high proportion of catch from coral-dependent species in the northern regions.

There was a weak trend towards regions with a higher per cent coral reef cover having a larger proportion of fishery catch for species that use live corals. The positive trend may reflect an overall greater productivity of coral reef species in regions with greater coral reef area. However, the trend was weak, and non-existent for reef-associated species, which reflects the multitude of other factors that are important contributors to catch. For example, reef-associated species, like various king prawn species (*Melicertus* spp.), may recruit to coral reefs, but will readily use other habitats in areas where reefs are absent (Table S5). The dependence of a region’s fisheries on reef species will also dependent on historical and socio-economic factors that were not analysed here, such as regional preferences for certain species e.g.^8^. Therefore, the general patterns discerned here for different categories are only an approximate guide to what might occur in other parts of the world.

We analysed stock status for six of the biggest reef-dependent species groups by catch. Red emperor and goldband snapper were found to be at the greatest risk of overfishing if there were declines in productivity across the entire GBR. Saddletail snapper, the species with the lowest productivity, was predicted to already be overfished. We are not aware of a current stock assessment for these species, however a major portion of the catch is likely taken by recreational fisheries. These stocks are also a minor portion of the line fishery, so reporting in logbooks may not be as consistent over time as for the major species. The status of these stock warrants further investigation. Coral trout, the major component of the line fishery catch, was sensitive to productivity declines of > 30%. Coral trout (2014^33^, 2019^46^) and red throat emperor (2006^47^) have been assessed in Queensland and both were found to be fished below their MSY levels. This gives these fisheries a buffer against productivity declines caused by habitat loss.

There are some limitations to our dynamic analysis, which may mean it provides a lower bound (optimistic) assessment of risk. The dynamic stock analysis did not consider recreational catches, because no time-series of recreational CPUE is available. Stock status estimates are not sensitive to consistent under-estimation of catch^48^. Our methodology is also simpler than that used in the stock assessments for red throat emperor and coral trout. For example, our model did not consider time-lags between recruitment and spawning. Despite these limitations, our quantitative assessments are consistent with the latest stock assessments where they were available, for instance our predictions for the biomass of coral trout relative to unfished biomass in 2017–2018 was similar to the latest stock assessment that used much more detailed data on spatial structure and fishing effort than we did^46^. We also note that our quantitative model likely underestimates the risk of overfishing given a decline in productivity. Further work could use age-structured stock assessment models to model the specific dependence of fish life-histories on coral structure, which may vary from changes in consumption and trophic level in adult fish^49^, to changes in recruitment of early life-stages^36^.

**Figure 6:**
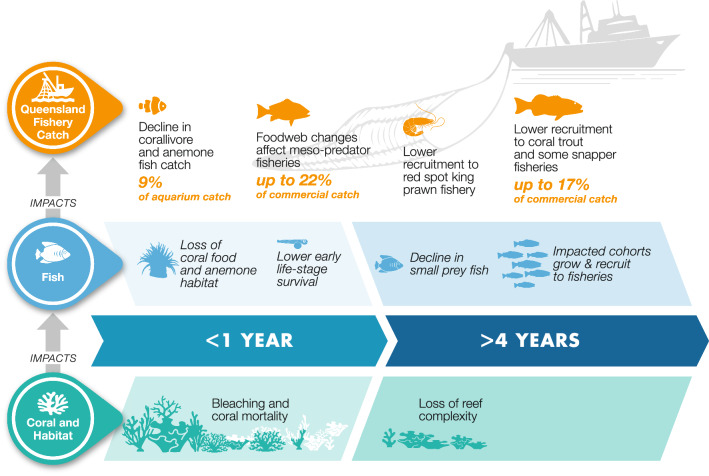
Summary of the main processes and time-lags by which coral bleaching may affect Queensland’s fisheries. Supporting references are in Table S5.

A stock assessment to revise the total allowable catch for reef fisheries would need quantitative estimates linking reef habitat loss to stock productivity parameters^12,46^. Current stock assessments have identified this knowledge gap as a major limitation^46^. Past stock assessments for coral trout have not found any statistical association between bleaching events and coral trout catchability^33^. This previous stock assessment did, however, observe that coral loss caused by cyclones correlated with lower catchability of coral trout over the following years^33^. It was hypothesised in the assessment that loss of structurally complex corals may make it easier for coral trout to hunt prey fish. It was further hypothesized that greater prey availability would mean that coral trout are less likely to take baited lines^33^, reducing the catchability of coral trout by the fishery is reduced. However, empirical food-web studies found reef degradation was detrimental to *Plectropomus maculatus* accessing prey^49^, and that this species has declined in biomass on degraded reefs^49,50^. The effects of reef degradation on foodweb interactions may also manifest as longer-term impacts than changes in catchability (Fig. 6), for instance by changing the overall productivity of prey species^27,51^. It is important to resolve the mechanisms through which fishery species respond to coral degradation because the different mechanisms suggest different fishery management responses to reef degradation. A decline in catchability would support a loosening of gear restrictions on the fishery to enable greater efficiency, whereas a decline in biomass would suggest tightening of catch limits to avoid recruitment overfishing in populations affected by reef degradation. Further, we need to know if these mechanisms vary by species, because findings from studies of *P. maculatus* may not apply to the fishery, which predominantly catches *P. leopardus*.

Complicating the detection of the effects of coral loss on fishery productivity is the expectation that catch of most targeted species will not respond directly to loss of live coral over timescales of < 5 years (Fig. 5, Table S5^27,51^). This lag occurs for several reasons. Loss of live coral cover may eventually lead to a loss in reef architectural complexity and complex habitats support refuges for settling and juvenile fish (e.g.^36,52^). Thus, the impact of coral loss on recruitment will not be detectable in fishery statistics until several years later when the cohort recruits to the fishery. Loss of habitat complexity may also restructure food-webs in ways that can enhance or diminish the overall productivity of fisheries^49,51^, so overall catch may be stabilized by targeting of multiple species^53^. Fishery independent time-series, such as from underwater visual surveys may therefore be important to combine with fishery dependent datasets to provide reliable warning of changes in the productivity of fisheries (e.g.^6,54^).

Fisheries productivity is susceptible to many other environmental impacts that may confound the effects of habitat loss. In particular, bleaching events are associated with marine heatwaves. The 2016 heatwave caused widespread shifts in reef fish communities^4^. Experimental evidence also suggests marine heatwaves affect escape responses, metabolism and feeding behaviour of target coral trout species^55,56^. Marine heatwaves can also have trophic impacts by increasing the bioenergetic cost of foraging, which negatively impacts reproduction and recruitment success^57^. We did not consider the susceptibility of reef fisheries to heatwaves, but future studies could do so using bioenergetics models (e.g.^58^).

### Habitat associations

While we identified habitat associations for all of the target species we included, there was little information on the quantitative effect of coral loss on population dynamics (Table S5). For instance, Spanish Mackerel > 40 cm can associate with reefs, including to use them as spawning sites^59^, yet there was little evidence for impacts of coral loss on their populations. Similarly, the sea cucumber fishery predominantly caught reef associated species, but there is only weak evidence for a dependence on corals^35^. In contrast, experimental studies indicate strong preference of sea cucumber larvae of some species towards settling near other tropical shallow water habitats, including seagrass^45^. Expanding these settlement studies to a greater number of coral reef fishery species may assist in quantifying the impacts of coral loss on fishery production. More generally, negative effects of coral bleaching are likely to be mitigated in areas where mangrove or seagrass co-occur and where species are also known to associate with these habitats^60^.

Our review quantified the portion of Queensland’s catch that depends on coral, but not the direction of the response to coral loss. Responses of fishery species to coral loss are diverse, with both increases and decreases in productivity, and changes in response direction over time e.g.^6,14,27,61^. This response diversity gives fisheries resilience to environmental shocks, through the portfolio effect^53^, though overall stability of catches may be eroded^6^. It is not clear whether the GBR’s line fishery would benefit from a portfolio effect, because the productivity trends observed in ref^6^ were herbivore fisheries, and herbivorous fish are not an important part of the GBR catch. Even the aquarium fishery, which hand captures live coral, may be able to adapt to bleaching, because it captures a range of species, including deeper species that may have variable responses to bleaching^28^. Therefore, management will need to know the likely direction of responses of fishery species to coral loss.

Future work should address how habitat indicators can be incorporated into stock assessments and management strategies. For instance, the coral and aquarium fisheries have a coral stress response plan that was developed as a collaboration between industry, government, NGO and scientists^32^. During years of strong bleaching, the Queensland industry body responsible for aquarium fishing voluntarily ceases harvesting of species most likely to be sensitive to bleaching, including certain anemonefish, anemone species, and corals, with the aim of supporting ecosystem recovery. Collectors are also contributing to monitoring ecosystem health at collection sites. This approach could be adopted across other sectors that may be sensitive to habitat loss. For instance, sensitivity to coral habitat loss, dependence on other habitats and their sensitivity to heatwaves or other sources of stresses and shocks could be incorporated into existing vulnerability assessments to help prioritise fisheries and species where revised management is needed^62^. Examples of collaborative response plans might include rotational closures that may help maintain fishery productivity^63^.

The sensitivity of fishery productivity to coral loss is likely to vary widely across tropical nations. Queensland may have lower sensitivity than other jurisdictions, because its catches are dominated by industrial fishing. Maritime nations where fishing is dominated by, and food and nutritional security as well as livelihoods dependent upon, artisanal and subsistence fisheries may have much greater sensitivity to coral loss^9^—although responses will depend on structure of fish assemblages and associated catch composition^6^. The protocol used here could be extended and applied to analyse catches in other countries and compare the degree to which catches depend on coral, as well as other inshore and offshore habitats.

Recent mass bleaching observations are in line with projections that such events will become a dominant and widespread driver of coral reef degradation^1^. The threat that rapid degradation from bleaching poses to the productivity of tropical fisheries is therefore of great contemporary management concern, and can be addressed through the reforms currently being undertaken for Queensland’s fisheries (e.g.^14^). This study found that, overall, coral reef bleaching poses a low risk to the habitats used by currently targeted Queensland fisheries species. However, catches by specific sectors and in given regions consisted of species with greater dependency on coral habitats, with some of these stocks likely susceptible to overfishing if reef habitat loss causes large declines in stock productivity. Ongoing research to identify the processes by which coral reef degradation impacts fisheries will help inform adaptive reef fisheries management responses to coral bleaching. Future assessments of Queensland’s fish catch should also expand scope to consider other impacts of climate change, like heatwaves and other environmental threats to coral reef fisheries, including water quality and cyclones.

## Supplementary information

Supplementary Information 1

## Data Availability

Data are freely available from Queensland Government Department of Fisheries and Agriculture via their Qfish site.

## References

[1] Hughes TP (2018). Spatial and temporal patterns of mass bleaching of corals in the Anthropocene. Science.

[2] Hoegh-Guldberg O (1999). Climate change, coral bleaching and the future of the world's coral reefs. Mar. Freshw. Res..

[3] Hughes TP (2018). Global warming transforms coral reef assemblages. Nature.

[4] Stuart-Smith RD, Brown CJ, Ceccarelli DM, Edgar GJ (2018). Ecosystem restructuring along the Great Barrier Reef following mass coral bleaching. Nature.

[5] Richardson LE, Graham NA, Pratchett MS, Eurich JG, Hoey AS (2018). Mass coral bleaching causes biotic homogenization of reef fish assemblages. Glob. Change Biol..

[6] Robinson JP (2019). Productive instability of coral reef fisheries after climate-driven regime shifts. Nat. Ecol. Evol..

[7] McClanahan T, Allison EH, Cinner JE (2015). Managing fisheries for human and food security. Fish Fish..

[8] Hanich Q (2018). Small-scale fisheries under climate change in the Pacific Islands region. Mar. Policy.

[9] Bell JD (2013). Mixed responses of tropical Pacific fisheries and aquaculture to climate change. Nat. Clim. Change.

[10] Sale, P. F. & Hixon, M. A. in *Interrelationships Between Corals and Fisheries* (ed S.A. Bortone) 7–18 (CRC Press, Boca Raton, 2015).

[11] Pratchett MS, Hoey AS, Wilson SK, Messmer V, Graham NA (2011). Changes in biodiversity and functioning of reef fish assemblages following coral bleaching and coral loss. Diversity.

[12] Brown CJ (2019). The assessment of fishery status depends on fish habitats. Fish Fish..

[13] Hughes TP (2017). Global warming and recurrent mass bleaching of corals. Nature.

[14] Pratchett MS (2017). Effects of climate change on coral grouper (Plectropomus spp) and possible adaptation options. Rev. Fish Biol. Fish..

[15] Schuerch M (2018). Future response of global coastal wetlands to sea-level rise. Nature.

[16] Cheal AJ, MacNeil MA, Emslie MJ, Sweatman H (2017). The threat to coral reefs from more intense cyclones under climate change. Glob. Change Biol..

[17] Wilson LJ (2016). Climate-driven changes to ocean circulation and their inferred impacts on marine dispersal patterns. Glob. Ecol. Biogeogr..

[18] Brown CJ (2017). Habitat change mediates the response of coral reef fish populations to terrestrial run-off. Mar. Ecol. Prog. Ser..

[19] Great Barrier Reef Foundation. www.barrierreef.org (2020).

[20] State of Queensland (2018). Reef 2050 Water Quality Improvement Plan 2017–2020.

[21] Jiddawi NS, Öhman MC (2002). Marine fisheries in Tanzania. Ambio.

[22] Nordlund LM, Unsworth RK, Gullström M, Cullen-Unsworth LC (2018). Global significance of seagrass fishery activity. Fish Fish..

[23] State of Queensland Department of Agriculture Fisheries and Forestry. *QFish data cube*. https://qfish.fisheries.qld.gov.au/ (2020).

[24] Taylor S, Webley J, McInnes K (2012). 2010 Statewide Recreational Fishing Survey.

[25] Anderson SC (2017). Benefits and risks of diversification for individual fishers. Proc. Natl. Acad. Sci..

[26] Fulton CJ (2020). Macroalgal meadow habitats support fish and fisheries in diverse tropical seascapes. Fish Fish..

[27] Graham NA (2007). Lag effects in the impacts of mass coral bleaching on coral reef fish, fisheries, and ecosystems. Conserv. Biol..

[28] Pratchett MS (2020). Bleaching susceptibility of aquarium corals collected across northern Australia. Coral Reefs.

[29] Delbeek, J. C. in *Biology of Butterflyfishes* (eds MS Pratchett, Michael L Berumen, & B Kapoor) 292–395 (CRC Press, Boca Raton, 2013).

[30] Wilson, S. K., Graham, N. A. & Pratchett, M. S. in *Biology of Butterflyfishes* (eds MS Pratchett, Michael L Berumen, & B Kapoor) 226–245 (CRC Press, Boca Raton, 2013).

[31] Beldade R, Blandin A, O’Donnell R, Mills SC (2017). Cascading effects of thermally-induced anemone bleaching on associated anemonefish hormonal stress response and reproduction. Nat. Commun..

[32] Donnelly R (2013). Stewardship Action Plan 2013: Mitigating Ecological Risk in a Changing Climate.

[33] Leigh GM, Campbell AB, Lunow CP, O'Neill MF (2014). Stock Assessment of the Queensland East Coast Common Coral Trout (Plectropomus leopardus) fishery.

[34] Russell D, Garrett R (1983). Use by juvenile barramundi, Lates calcarifer (Bloch), and other fishes of temporary supralittoral habitats in a tropical estuary in northern Australia. Mar. Freshw. Res..

[35] Eriksson H, Fabricius-Dyg J, Lichtenberg M, Perez-Landa V, Byrne M (2010). Biology of a high-density population of Stichopus herrmanni at One Tree Reef, Great Barrier Reef, Australia. SPC Beche-de-mer Information Bulletin.

[36] Wen C, Pratchett M, Almany G, Jones G (2013). Patterns of recruitment and microhabitat associations for three predatory coral reef fishes on the southern Great Barrier Reef, Australia. Coral Reefs.

[37] Graham NAJ (2011). Extinction vulnerability of coral reef fishes. Ecol. Lett..

[38] DNRME. Reefs and Shoals—Queensland. (2018).

[39] Rue H, Martino S, Chopin N (2009). Approximate Bayesian inference for latent Gaussian models by using integrated nested Laplace approximations. J. R. Stat. Soc. Ser. B (Stat. Methodol.).

[40] Teixeira D, Zischke MT, Webley JA (2016). Investigating bias in recreational fishing surveys: Fishers listed in public telephone directories fish similarly to their unlisted counterparts. Fish. Res..

[41] Webley J, McInnes K, Teixeira D, Lawson A, Quinn R (2015). 2014 Statewide Recreational Fishing Survey.

[42] Hilborn R (2020). Effective fisheries management instrumental in improving fish stock status. Proc. Natl. Acad. Sci..

[43] Millar RB, Meyer R (2000). Non-linear state space modelling of fisheries biomass dynamics by using Metropolis-Hastings within-Gibbs sampling. J. Roy. Stat. Soc. Ser. C (Appl. Stat.).

[44] Meynecke J-O, Lee SY, Duke NC, Warnken J (2006). Effect of rainfall as a component of climate change on estuarine fish production in Queensland, Australia. Estuar. Coast. Shelf Sci..

[45] Mercier A, Battaglene SC, Hamel J-F (2000). Settlement preferences and early migration of the tropical sea cucumber Holothuria scabra. J. Exp. Mar. Biol. Ecol..

[46] Campbell, A., Leigh, G., Bessel-Browne, P. & Lovett, R. Stock assessment of the Queensland east coast common coral trout (Plectropomus leopardus) fishery. April 2019. (State of Queensland., Brisbane, 2019).

[47] Leigh G, Williams A, Begg G, Gribble N, Whybird O (2006). Stock Assessment of the Queensland East Coast Red Throat Emperor (*Lethrinus miniatus*), Queensland Department of Primary Industries and Fisheries, Brisbane.

[48] Rudd MB, Branch TA (2017). Does unreported catch lead to overfishing?. Fish Fish..

[49] Hempson TN (2017). Coral reef mesopredators switch prey, shortening food chains, in response to habitat degradation. Ecol. Evol..

[50] Williamson DH, Ceccarelli DM, Evans RD, Jones GP, Russ GR (2014). Habitat dynamics, marine reserve status, and the decline and recovery of coral reef fish communities. Ecol. Evol..

[51] Rogers A, Blanchard JL, Mumby PJ (2018). Fisheries productivity under progressive coral reef degradation. J. Appl. Ecol..

[52] Graham N, Nash K (2013). The importance of structural complexity in coral reef ecosystems. Coral Reefs.

[53] Robinson JP (2020). Diversification insulates fisher catch and revenue in heavily exploited tropical fisheries. Sci. Adv..

[54] Edgar GJ, Ward TJ, Stuart-Smith RD (2018). Rapid declines across Australian fishery stocks indicate global sustainability targets will not be achieved without an expanded network of ‘no-fishing’reserves. Aquat. Conserv. Mar. Freshw. Ecosyst..

[55] Johansen J (2015). Large predatory coral trout species unlikely to meet increasing energetic demands in a warming ocean. Sci. Rep..

[56] Clark TD, Messmer V, Tobin AJ, Hoey AS, Pratchett MS (2017). Rising temperatures may drive fishing-induced selection of low-performance phenotypes. Sci. Rep..

[57] Brodeur RD, Hunsicker ME, Hann A, Miller TW (2019). Effects of warming ocean conditions on feeding ecology of small pelagic fishes in a coastal upwelling ecosystem: a shift to gelatinous food sources. Mar. Ecol. Prog. Ser..

[58] Krieger JR, Beaudreau AH, Heintz RA, Callahan MW (2020). Growth of young-of-year sablefish (Anoplopoma fimbria) in response to temperature and prey quality: insights from a life stage specific bioenergetics model. J. Exp. Mar. Biol. Ecol..

[59] Ryan, S. Ecological assessment of the Queensland East Coast Spanish Mackerel fishery. 103 (Queensland Government, Department of Primary Industries and Fisheries, Brisbane, Queensland, 2004).

[60] Rogers A, Mumby PJ (2019). Mangroves reduce the vulnerability of coral reef fisheries to habitat degradation. PLoS Biol..

[61] Morais RA, Bellwood DR (2019). Pelagic subsidies underpin fish productivity on a degraded coral reef. Curr. Biol..

[62] Dee L, Karr K, Landesberg C, Thornhill D (2019). Assessing vulnerability of fish in the U.S. marine aquarium trade. Front. Mar. Sci..

[63] Plagányi ÉE, Skewes T, Murphy N, Pascual R, Fischer M (2015). Crop rotations in the sea: Increasing returns and reducing risk of collapse in sea cucumber fisheries. Proc. Natl. Acad. Sci..

