# Supplementary material for: Dependency of Queensland and the Great Barrier Reef’s tropical fisheries on reef-associated fish
Source: Sci Rep. 2020 Oct 20;10:17801. doi: 10.1038/s41598-020-74652-2 (PMC7576786; doi:10.1038/s41598-020-74652-2)
Supplement: Supplementary file 1 — Supplementary Information 1 [file 41598_2020_74652_MOESM1_ESM.docx]

Dependency of Queensland and the Great Barrier Reef’s tropical fisheries on reef-associated fish

**Authors:** Christopher J. Brown^1*^, William Taylor^1^, Colette C. Wabnitz^2,3^, Rod M. Connolly^4^

**Affiliations**

1. Australian Rivers Institute – Coast and Estuaries, School of Environment and Science, Griffith University, Nathan, QLD, 4111, Australia

2. Institute for the Oceans and Fisheries, The University of British Columbia, 2202 Main Mall, Vancouver, BC, Canada V6T1Z4

3. Center for Ocean Solutions, 473 Via Ortega, Stanford University, Stanford, CA 94305, United States

4. Australian Rivers Institute – Coast and Estuaries, School of Environment and Science,

Griffith University, Gold Coast, QLD, 4222, Australia

*Corresponding author: [chris.brown@griffith.edu.au](mailto:chris.brown@griffith.edu.au)

**Supplementary Material**

*Further details of catch data processing*

For the commercial fishery the data from ‘fish trawl’ (otter trawl and Danish Seine primarily targeting *Sillago robusta*) was omitted from analyses because species data for this fishery is incomplete, due to data privacy constraints. However, fishery status reports indicate the sector primarily targets *Sillago robusta*^1^.

For the sea cucumber fishery catches reported as ‘Curryfish’ were assumed to belong to genus *Stichopus*. While primary literature was not available for habitat use by all species individually (including *Actinopyga lecanora, Actinopyga miliaris, Actinopyga spinea, Bohadschia graeffei, Bohadschia marmorata, Stichopus vastus, Thelenota ananas, Thelenota anax*), general habitat preferences were extracted from a recent review study^2^.

The data on recreational fishery catch come from a randomised phone call survey. Recreational fishers are identified through phone calls and asked if they would like to sign up to a logbook program^3^. The sampling is stratified so that it is not biased towards a particular demographic. Volunteers then receive several follow up phone calls over a year’s duration and the scientists question them on when and where they went fishing and what they caught. Data are recorded as the number of fish caught (in contrast to the commercial data, which is biomass), by region. The regions are similar but not identical to regions used for reporting commercial catch^3^. Invertebrates were removed from the recreational fish catch, because catch numbers were inflated by large catches of prawns. Freshwater catches were also omitted from analyses.

The Queensland commercial fisheries data has two important caveats. Data for region or grids with less than five boats fishing in them are unavailable due to privacy restrictions on data use. This potential bias away from lightly fished areas should primarily affect the mapping of line catches. Because data are fishery-dependent, the adaptive responses of fishers to changes in availability of their target species may stabilise CPUE relative to real abundance changes. Despite these caveats, data were considered appropriate for analyses because: (1) there have been no recent changes to fishery management that would be expected to contribute to widespread bias in reporting; (2) they focussed on comparison of regional and sector trends relative to total reported catches.

*Methods for estimating the risk of overfishing in reef fisheries subject to sudden productivity declines*

We fitted Pella-Tomlinson surplus production models to CPUE time-series for the coral associated species with the largest catches (Table S1). We excluded mackerel fisheries because there was little likelihood that they would be impacted by loss of live corals. Other species had insufficient data for analysis. Surplus production models followed the Bayesian estimation framework of Millar and Myer^4^ and Winker et al.^5^. In this framework biomass in a year is estimated as a latent state from CPUE and catch time-series. We specified a strongly informative priors for parameter *r* (intrinsic growth rate) and weakly informative priors for *K* (carrying capacity), sigma (process errors for biomass) and tau (observation errors for CPUE), which facilitated identification of model parameters^4^. We assumed the shape parameter for the Pella-Tomlinson took a value so that BMSY/K = 0.4, as per global meta-analyses^6^.

We estimated two catchability parameters, one for 2004 and before and one for after 2004. 2004 was the year that a major restructure of the line fishery occurred coinciding with an increase in protected area coverage. Both changes may have affected catchability of coral trout by changing the spatial distribution of fishing and encouraging less efficient fishers to leave^7^.

The biomass in the initial year of simulation (1990) is critical for model estimation. We assumed biomass that was 80% of K in 1990 for all stocks. The standard deviation of the prior for ln *K* was set assuming a coefficient of variation of 50%. Commercial line fisheries date back to the 1950s, but depletion was likely limited before the 1990s. In the 1990s export to Asian markets began and catches peaked^7^. Further, initial model investigation indicated that an 80% initial depletion resulted in ~20% depletion of coral trout by 2004, which is consistent with earlier modelling and empirical studies^8^. We lacked data for other species so we assumed the same depletion history.

The prior for *r* is critical^4^, it must be sufficiently constrained for estimation because the *r*  parameter is partly confounded with the *K*  parameter. We used a log-normal prior for *r.* We estimated the mean of the prior for *r* from the parameters in the meta-analysis of Thorson 2020^9^, using a coefficient of variation of 10% to set the standard deviation. Other priors were set to be weakly informative to facilitate efficient model estimation^4^. After some experimentation we came to the values in Table S2, priors with broader quantiles than those in Table S2 had negligible impact on the estimated parameters but slowed convergence of the MCMC chains.

Reporting of several species groups has changed over time, therefore we followed Queensland fisheries stock assessment protocol by running assessments on mixed species groups for: goldband snapper group (*Pristipomoides multidens and typus,* including CAAB codes 37346901, 37346019, 37346002) and red spot king prawns (*Melicertus longistylus* catches dominated by that species, includes CAAB codes 28711048, 28711910, 28711908).

A further uncertainty in the data is the standardization of effort. There is evidence of power increases in the trawl fishery^10^, which would increase the catchability parameter and potentially cause CPUE hyper-stability. We are not aware of any quantitative studies of how fishing efficiency has changed over time in the line fishery, though it is well known that efficiency varies by fishing practices (e.g. number of dories), operators and regions^11^. Efficiency may have increased through the 1990s with increasing prevalence of chart plotters and view buckets^11^. Efficiency also likely changed with the 2004 restructure and after, though it is not clear in what direction. The restructure may have forced less efficient fishers to leave the fishery, but also the rezoning spatially constrained fishing practices and affected ability to find and target catch hotpots^11^. Economic changes in the fishery since 2004 may have also affected the efficiency^11^. We therefore ran a sensitivity assuming a 1% increase in fishing power per annum. We ran higher rates of increase (global averages are as high as 4% p.a.), but they were unrealistic in that all stocks were predicted to be overfished in the present day.

Models were fit using the Stan program for Bayesian computing^12^. We used 3 chains, a tree depth of 12 and took 500 samples for burnin and 4500 samples for estimation. We did the standard MCMC diagnostic checks for convergence and confirmed all Rhat statistics were near 1.

**Table S1** Parameters used for each of the species in the analysis of stock status.

| Species group | Maximum age | Age at maturity | Mortality rate | Estimate for r | Assumed initial depletion |
| --- | --- | --- | --- | --- | --- |
| Coral trout | 18 | 4 | 0.3 | 0.43 | 80% |
| Redthroat emperor | 18 | 5 | 0.45 | 0.42 | 80% |
| Saddletail snapper | 18 | 4 | 0.2 | 0.38 | 80% |
| Red Emperor | 25 | 5 | 0.2 | 0.28 | 80% |
| Goldband snapper | 16 | 3 | 0.3 | 0.57 | 80% |
| Red spot king prawn | 3 | 4 months | 2.4 | 1.4 | 80% |

**Table S2** Priors and their distributions for the surplus production models

| Parameter | Prior distribution and parameters | Derivation of parameters |
| --- | --- | --- |
| Carrying capacity | Log normal(Kmean, Ksd) | Kmean set assuming biomass in the first year was 80% of K  Ksd set assuming a coefficient of variation of 50% |
| Intrinsic growth rate | Log normal(rmean, rsd) | rmean as in table S1,  rsd was set assuming a coefficient of variation of 10% |
| catchability (natural log) | Uniform(0.01, 5) |  |
| Standard deviation of the latent biomass variable | Gamma(shape = 5, rate = 0.15) |  |
| Standard deviation of the CPUE | Gamma(shape = 6, rate = 0.5) |  |

**Table S3** R^2^ goodness of fit statistics for the surplus production models fitted to each species, assuming either stable or increasing fishery power.

| Group name | Power increase? | CPUE R^2^ |
| --- | --- | --- |
| Coral trout | +1% p.a. | 0.98 |
| Coral trout | Stable | 0.98 |
| Goldband snapper | +1% p.a. | 0.97 |
| Goldband snapper | Stable | 0.96 |
| Red Emperor | +1% p.a. | 0.99 |
| Red Emperor | Stable | 0.98 |
| Redthroat Emperor | +1% p.a. | 0.99 |
| Redthroat Emperor | Stable | 0.98 |
| RSK prawn | +1% p.a. | 0.81 |
| RSK prawn | Stable | 0.82 |
| Saddletail Snapper | +1% p.a. | 0.97 |
| Saddletail Snapper | Stable | 0.98 |

**Table S4** Model parameters from surplus production models with means and 89% credible intervals. Where K is carrying capacity lnq1 is the natural log of the catchability before the restructure, lnq2 is the natural log of the catchability after the restructure, r is the intrinsic growth parameter, sigma is the standard deviation of logged biomass estimates and tau is the standard deviation of logged CPUE.

| Group name | Fishery power increase? | Parameter | Mean | Lower 0.89% | Upper 0.89% |
| --- | --- | --- | --- | --- | --- |
| coral trout | +1% p.a. | K | 32.87 | 25.84 | 39.00 |
| coral trout | Stable | K | 35.30 | 28.81 | 42.29 |
| coral trout | +1% p.a. | lnq1 | 0.52 | 0.28 | 0.77 |
| coral trout | Stable | lnq1 | 0.49 | 0.25 | 0.71 |
| coral trout | +1% p.a. | lnq2 | 0.22 | 0.01 | 0.44 |
| coral trout | Stable | lnq2 | 0.28 | 0.02 | 0.52 |
| coral trout | +1% p.a. | r | 0.40 | 0.33 | 0.46 |
| coral trout | Stable | r | 0.39 | 0.33 | 0.45 |
| coral trout | +1% p.a. | sigma | 0.14 | 0.09 | 0.19 |
| coral trout | Stable | sigma | 0.12 | 0.08 | 0.17 |
| coral trout | +1% p.a. | tau | 0.10 | 0.05 | 0.15 |
| coral trout | Stable | tau | 0.08 | 0.04 | 0.13 |
| Goldband snapper | +1% p.a. | K | 15.61 | 12.46 | 18.63 |
| Goldband snapper | Stable | K | 16.96 | 13.60 | 19.91 |
| Goldband snapper | +1% p.a. | lnq1 | 0.21 | 0.01 | 0.39 |
| Goldband snapper | Stable | lnq1 | 0.19 | 0.01 | 0.36 |
| Goldband snapper | +1% p.a. | lnq2 | 0.21 | 0.01 | 0.42 |
| Goldband snapper | Stable | lnq2 | 0.19 | 0.01 | 0.39 |
| Goldband snapper | +1% p.a. | r | 0.53 | 0.44 | 0.61 |
| Goldband snapper | Stable | r | 0.54 | 0.45 | 0.62 |
| Goldband snapper | +1% p.a. | sigma | 0.27 | 0.18 | 0.36 |
| Goldband snapper | Stable | sigma | 0.26 | 0.17 | 0.36 |
| Goldband snapper | +1% p.a. | tau | 0.17 | 0.08 | 0.26 |
| Goldband snapper | Stable | tau | 0.18 | 0.09 | 0.27 |
| Red Emperor | +1% p.a. | K | 29.00 | 20.83 | 36.18 |
| Red Emperor | Stable | K | 29.35 | 21.16 | 36.61 |
| Red Emperor | +1% p.a. | lnq1 | 0.56 | 0.27 | 0.83 |
| Red Emperor | Stable | lnq1 | 0.53 | 0.21 | 0.80 |
| Red Emperor | +1% p.a. | lnq2 | 0.22 | 0.01 | 0.53 |
| Red Emperor | Stable | lnq2 | 0.25 | 0.01 | 0.50 |
| Red Emperor | +1% p.a. | r | 0.25 | 0.21 | 0.29 |
| Red Emperor | Stable | r | 0.26 | 0.22 | 0.30 |
| Red Emperor | +1% p.a. | sigma | 0.23 | 0.15 | 0.29 |
| Red Emperor | Stable | sigma | 0.20 | 0.13 | 0.27 |
| Red Emperor | +1% p.a. | tau | 0.11 | 0.05 | 0.18 |
| Red Emperor | Stable | tau | 0.13 | 0.07 | 0.20 |
| Redthroat Emperor | +1% p.a. | K | 21.36 | 17.02 | 24.85 |
| Redthroat Emperor | Stable | K | 22.56 | 18.91 | 26.41 |
| Redthroat Emperor | +1% p.a. | lnq1 | 0.54 | 0.34 | 0.75 |
| Redthroat Emperor | Stable | lnq1 | 0.51 | 0.31 | 0.70 |
| Redthroat Emperor | +1% p.a. | lnq2 | 0.15 | 0.01 | 0.31 |
| Redthroat Emperor | Stable | lnq2 | 0.14 | 0.01 | 0.28 |
| Redthroat Emperor | +1% p.a. | r | 0.39 | 0.32 | 0.45 |
| Redthroat Emperor | Stable | r | 0.39 | 0.33 | 0.45 |
| Redthroat Emperor | +1% p.a. | sigma | 0.14 | 0.09 | 0.19 |
| Redthroat Emperor | Stable | sigma | 0.11 | 0.07 | 0.16 |
| Redthroat Emperor | +1% p.a. | tau | 0.09 | 0.05 | 0.13 |
| Redthroat Emperor | Stable | tau | 0.08 | 0.02 | 0.12 |
| RSK prawn | +1% p.a. | K | 12.05 | 9.92 | 14.15 |
| RSK prawn | Stable | K | 14.44 | 11.95 | 16.71 |
| RSK prawn | +1% p.a. | lnq1 | 0.34 | 0.10 | 0.58 |
| RSK prawn | Stable | lnq1 | 0.21 | 0.01 | 0.39 |
| RSK prawn | +1% p.a. | lnq2 | 0.12 | 0.01 | 0.23 |
| RSK prawn | Stable | lnq2 | 0.13 | 0.01 | 0.27 |
| RSK prawn | +1% p.a. | r | 1.32 | 1.12 | 1.52 |
| RSK prawn | Stable | r | 1.31 | 1.12 | 1.50 |
| RSK prawn | +1% p.a. | sigma | 0.18 | 0.09 | 0.27 |
| RSK prawn | Stable | sigma | 0.16 | 0.08 | 0.24 |
| RSK prawn | +1% p.a. | tau | 0.21 | 0.12 | 0.30 |
| RSK prawn | Stable | tau | 0.19 | 0.12 | 0.27 |
| Saddletail Snapper | +1% p.a. | K | 12.75 | 10.21 | 15.98 |
| Saddletail Snapper | Stable | K | 14.38 | 10.48 | 18.05 |
| Saddletail Snapper | +1% p.a. | lnq1 | 0.83 | 0.53 | 1.13 |
| Saddletail Snapper | Stable | lnq1 | 0.68 | 0.37 | 1.00 |
| Saddletail Snapper | +1% p.a. | lnq2 | 0.39 | 0.01 | 0.65 |
| Saddletail Snapper | Stable | lnq2 | 0.32 | 0.01 | 0.63 |
| Saddletail Snapper | +1% p.a. | r | 0.36 | 0.32 | 0.42 |
| Saddletail Snapper | Stable | r | 0.37 | 0.30 | 0.42 |
| Saddletail Snapper | +1% p.a. | sigma | 0.32 | 0.22 | 0.40 |
| Saddletail Snapper | Stable | sigma | 0.27 | 0.19 | 0.37 |
| Saddletail Snapper | +1% p.a. | tau | 0.19 | 0.10 | 0.28 |
| Saddletail Snapper | Stable | tau | 0.18 | 0.08 | 0.27 |

##

**
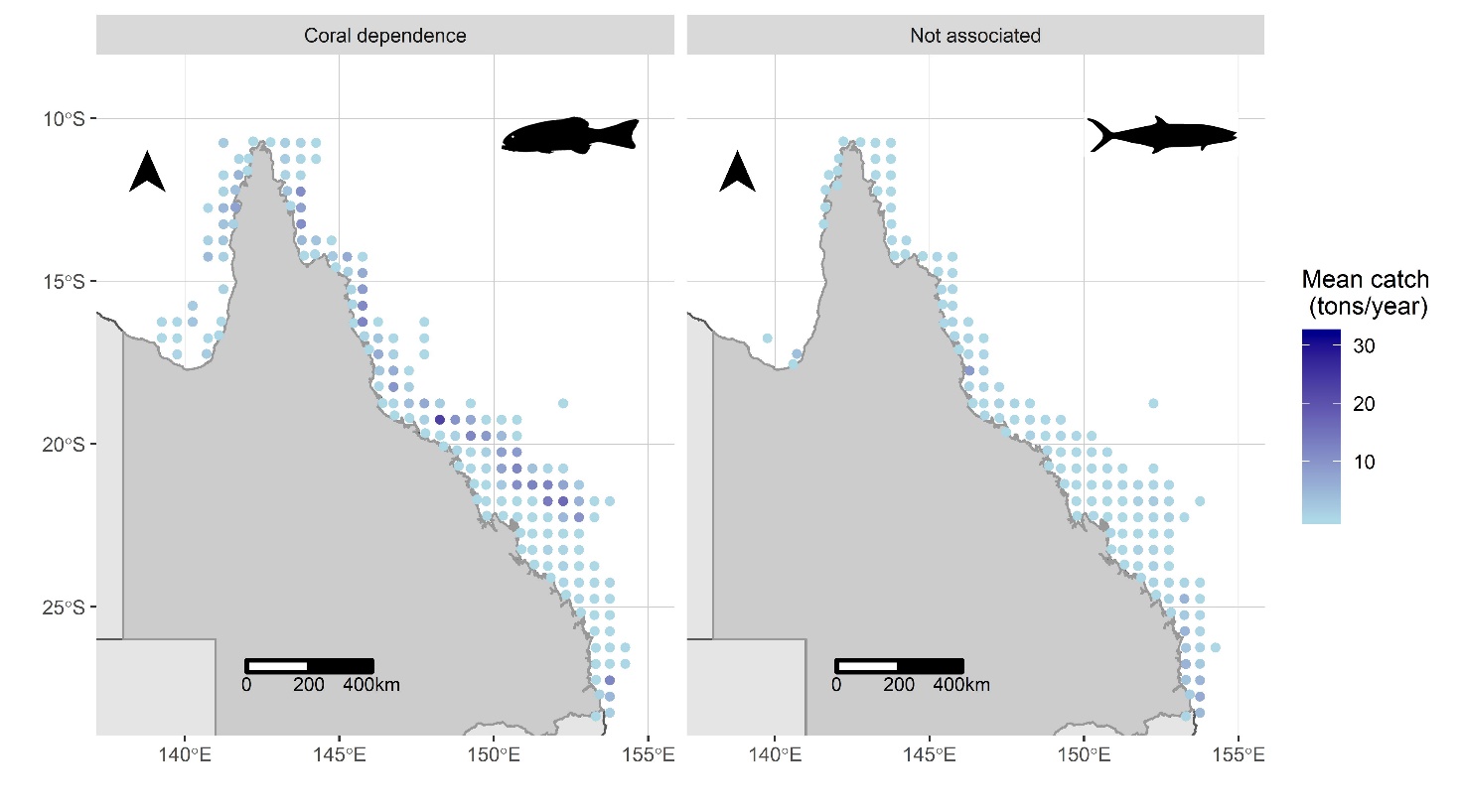
**

**Figure S1** Geographical distribution of catches (in tonnes) by the line fishing fleet for species associated with corals (a) and those not associated with corals (b) along the Queensland coast. The catch of species with dependence on live coral cover (category 3) and associating with corals (category 2) was grouped into the ‘coral dependence’ group for visualisation purposes. Each circle represents the centre of a Queensland fisheries statistical grid and darker circles indicate greater catches. Only grids with catch >0 are shown. The map of Queensland is from the GEODATA COAST 100K 2004 provided by Commonwealth of Australia (Geoscience Australia 2004), distributed under a Creative Commons Attribution 4.0 International Licence.


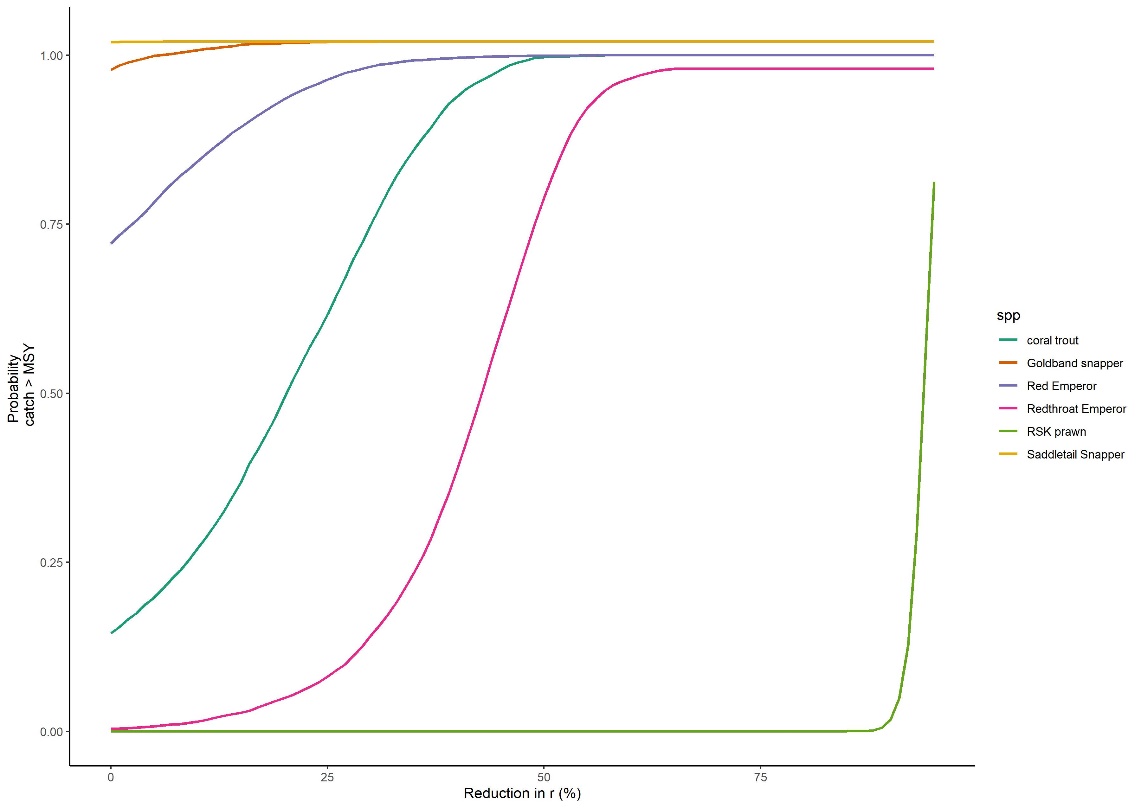


**Figure S2** Probability present day catch exceeds estimated MSY reference point given different assumed % reductions in the intrinsic growth rate (r). Models were fit to CPUE data assuming a 1% increase in fishing efficiency per year.

**Table S5** Expected impacts of coral reef mortality and loss of architectural complexity for on some of Queensland’s key fishery species, based on a literature review.

| **Species group** | **Fishery** | **Process of impact** | **Time-span to impacts on fishery catch** | **Expected impact on fishery** | **References** |
| --- | --- | --- | --- | --- | --- |
| Reef associated finfish, primarily coral trout, but also lutjanids, serranids, lethrinids | Primarily commercial line and recreational. Experimental trawl fishery in the Gulf of Carpentaria. | Live and structurally complex corals are larval settlement habitat and provide refuges from predation | >5 years given time for loss of architectural complexity and then recruitment lags | Declining productivity | Wen, et al. ^13,^Graham, et al. ^14,^Rogers, et al. ^15^ |
|  |  | Food-web impacts- loss of habitat complexity changes availability of prey. | >5 years, as above | Increasing or declining productivity | Rogers, et al. ^15,^Hempson, et al. ^16^ |
|  |  | Direct effects of temperature on swimming performance and trophic interactions | Immediate | Predicted declining productivity | Johansen, et al. ^17,^Pratchett, et al. ^18^ |
| Mixed reef fish particularly smaller-sized fish | Aquarium collection | Many species are dependent on corals or structurally complex habitats and some feed directly on corals | Immediate-5 years, loss of coral as a food source + eventual decline in complexity | Declining availability for collection | Richardson, et al. ^19,^Stuart-Smith, et al. ^20,^e.g. Matis, et al. ^21^ |
| Anemonefish | Aquarium collection | Anemones are subject to bleaching which stresses fish and causes decline in fecundity | <1 years based on immediate loss of anemones and then time lag to recruitment | Declining productivity | Beldade, et al. ^22,^Saenz-Agudelo, et al. ^23^ |
| Coral collection fisheries | Aquarium collection | Direct bleaching and mortality, ecosystem change | Immediate | Declining availability for collection | e.g. Hughes, et al. ^24^ |
| Tropical Rock Lobster | Diving | Dependent on structurally complex habitats for shelter | Unknown. Likely >5 years given time for loss of reef complexity | Declining availability for collection | Dennis, et al. ^25^ |
|  | Diving | Changes in growth, migration and mortality under warming | >10 years | Productivity may change, unclear in which direction | Plagányi, et al. ^26^ |
| Sea cucumbers | Hand capture | Commonly associated with coral reefs | Unclear | Unclear | Purcell, et al. ^27^ |
|  | Hand capture | Low spawning biomass for some species may mean they are vulnerable to changing environmental conditions | Unclear | Declining productivity | Johnson and Welch ^28^ |
| *Melicertus longistylus,* (red spot king prawn) and possibly also *Melicertus plebejus* (eastern king prawn) in areas that have coral reefs. | Otter trawl | Juveniles recruit to coral flat and lagoon areas, so loss of habitat could affect recruitment | >5 years based on time lags for loss of complexity and then declining recruitment 1 year after spawning | Declining productivity | Gribble, et al. ^29,^Courtney, et al. ^30^ |

**References**

1 Department of Agriculture and Fisheries. Status Report: Fin Fish (Stout Whiting) Trawl Fishery. (Queensland Government, Brisbane, Queensland, 2018).

2 Purcell, S., Samyn, Y. & Conand, C. Commercially Important Sea Cucumbers of the World. 150 (FAO, Rome, Italy: , 2012).

3 Taylor, S., Webley, J. & McInnes, K. 2010 Statewide Recreational Fishing Survey. (Queensland Government, Brisbane, Queensland, 2012).

4 Millar, R. B. & Meyer, R. Non‐linear state space modelling of fisheries biomass dynamics by using Metropolis‐Hastings within‐Gibbs sampling. *Journal of the Royal Statistical Society: Series C (Applied Statistics)* **49**, 327-342 (2000).

5 Winker, H., Carvalho, F. & Kapur, M. JABBA: just another Bayesian biomass assessment. *Fisheries Research* **204**, 275-288 (2018).

6 Thorson, J. T., Cope, J. M., Branch, T. A. & Jensen, O. P. Spawning biomass reference points for exploited marine fishes, incorporating taxonomic and body size information. *Canadian Journal of Fisheries and Aquatic Sciences* **69**, 1556-1568 (2012).

7 Leigh, G. M., Campbell, A. B., Lunow, C. P. & O'Neill, M. F. Stock assessment of the Queensland east coast common coral trout (Plectropomus leopardus) fishery. (Queensland Government, Brisbane, Queensland, 2014).

8 Hopf, J. K., Jones, G. P., Williamson, D. H. & Connolly, S. R. Synergistic effects of marine reserves and harvest controls on the abundance and catch dynamics of a coral reef fishery. *Current Biology* **26**, 1543-1548 (2016).

9 Thorson, J. T. Predicting recruitment density dependence and intrinsic growth rate for all fishes worldwide using a data‐integrated life‐history model. *Fish and Fisheries* **21**, 237-251 (2020).

10 O’Neill, M. F. & Leigh, G. M. Fishing power increases continue in Queensland's east coast trawl fishery, Australia. *Fisheries Research* **85**, 84-92 (2007).

11 Leigh, G., Williams, A., Begg, G., Gribble, N. & Whybird, O. Stock assessment of the Queensland east coast Red Throat Emperor (Lethrinus miniatus), Queensland Department of Primary Industries and Fisheries, Brisbane. (Queensland Government, Brisbane, Queensland, 2006).

12 Carpenter, B. *et al.* Stan: A probabilistic programming language. *Journal of statistical software* **76** (2017).

13 Wen, C. K. *et al.* Recruitment hotspots boost the effectiveness of no-take marine reserves. *Biological Conservation* **166**, 124-131, doi:10.1016/j.biocon.2013.06.017s (2013).

14 Graham, N. A. *et al.* Lag effects in the impacts of mass coral bleaching on coral reef fish, fisheries, and ecosystems. *Conservation Biology* **21**, 1291-1300 (2007).

15 Rogers, A., Blanchard, J. L. & Mumby, P. J. Fisheries productivity under progressive coral reef degradation. *Journal of Applied Ecology* **55**, 1041-1049 (2018).

16 Hempson, T. N. *et al.* Coral reef mesopredators switch prey, shortening food chains, in response to habitat degradation. *Ecology and Evolution* **7**, 2626-2635 (2017).

17 Johansen, J. *et al.* Large predatory coral trout species unlikely to meet increasing energetic demands in a warming ocean. *Scientific Reports* **5**, 13830 (2015).

18 Pratchett, M. S. *et al.* Effects of climate change on coral grouper (Plectropomus spp.) and possible adaptation options. *Reviews in Fish Biology and Fisheries* **27**, 297-316 (2017).

19 Richardson, L. E., Graham, N. A., Pratchett, M. S., Eurich, J. G. & Hoey, A. S. Mass coral bleaching causes biotic homogenization of reef fish assemblages. *Global Change Biology* **24**, 3117-3129 (2018).

20 Stuart-Smith, R. D., Brown, C. J., Ceccarelli, D. M. & Edgar, G. J. Ecosystem restructuring along the Great Barrier Reef following mass coral bleaching. *Nature* **560**, 92-96 (2018).

21 Matis, P. A., Donelson, J. M., Bush, S., Fox, R. J. & Booth, D. J. Temperature influences habitat preference of coral reef fishes: Will generalists become more specialised in a warming ocean? *Global Change Biology* **24**, 3158-3169 (2018).

22 Beldade, R., Blandin, A., O’Donnell, R. & Mills, S. C. Cascading effects of thermally-induced anemone bleaching on associated anemonefish hormonal stress response and reproduction. *Nature Communications* **8**, 716 (2017).

23 Saenz-Agudelo, P., Jones, G., Thorrold, S. & Planes, S. Detrimental effects of host anemone bleaching on anemonefish populations. *Coral Reefs* **30**, 497-506 (2011).

24 Hughes, T. P. *et al.* Global warming transforms coral reef assemblages. *Nature* **556**, 492 (2018).

25 Dennis, D., Skewes, T. & Pitcher, C. Habitat use and growth of juvenile ornate rock lobsters, Panulirus ornatus (Fabricius, 1798), in Torres Strait, Australia. *Marine and Freshwater Research* **48**, 663-670 (1997).

26 Plagányi, É. E. *et al.* Assessing the adequacy of current fisheries management under changing climate: a southern synopsis. *ICES Journal of Marine Science* **68**, 1305-1317, doi:10.1093/icesjms/fsr049 (2011).

27 Purcell, S. W., Conand, C., Uthicke, S. & Byrne, M. in *Oceanography and marine biology* 375-394 (CRC Press, 2016).

28 Johnson, J. E. & Welch, D. J. Climate change implications for Torres Strait fisheries: assessing vulnerability to inform adaptation. *Climatic Change* **135**, 611-624 (2016).

29 Gribble, N., Wassenberg, T. & Burridge, C. Factors affecting the distribution of commercially exploited penaeid prawns (shrimp)(Decapod: Penaeidae) across the northern Great Barrier Reef, Australia. *Fisheries research* **85**, 174-185 (2007).

30 Courtney, A. *et al.* Biological and economic management strategy evaluations of the eastern king prawn fishery. *Project Report. FRDC.* (2014).
